# Safeguarding governance and advancing policy at the nexus of climate and health: a commercial determinants of health perspective

**DOI:** 10.1016/j.joclim.2025.100610

**Published:** 2026-01-12

**Authors:** Daniel Hunt, Britta K Matthes

**Affiliations:** a Centre for 21st Century Public Health, Department for Health, University of Bath, Bath, United Kingdom; b School of Public Health, The University of Queensland, Brisbane, Queensland, Australia

**Keywords:** Policy, Health, Health equity, Commercial determinants of health, Climate change, Governance

## Abstract

The impacts of climate change on physical and mental health and health equity are well-established. Intersectoral and multisectoral policy responses are emerging, yet current entry-points, to build decarbonised and climate-resilient health services or respond to health emergencies, risk trapping health in downstream and biomedical policy paradigms at the expense of wider determinants. However, climate change does not only undermine health and equity by worsening disease pathways and burdens. Drawing on a wide range of literature, this perspective deepens our understanding, exploring how climate change destabilizes key political, economic, social and technological systems necessary to govern for health and equity, and why this is a public health concern. Using a ‘commercial determinants of health’ lens, we then investigate how commercial activities misaligned with health can cause, worsen and exploit destabilized conditions of governance, and how climate change undermines health-enabling commercial ecosystems. Learning from examples across industries and sectors, four recommendations are made. First, governments should cooperate to mainstream determinants of health and health equity approaches in intersectoral and multisectoral health and climate policies, locally to globally. Second, governments should prioritize policies that explicitly address commercial activities as determinants of climate change and health. Third, governments and international finance institutions (IFIs) should acknowledge their past decisions have accelerated climate change as an existential threat to sustainable economic development, and urgently re-evaluate normative approaches to market externalities, climate financing and human and institutional capital development. Fourth, policymakers have opportunities to champion untapped co-benefits of resilient economies, sustainable development, human health and planetary flourishing.

## Highlights

• Climate change is destabilizing systems of governance for health and health equity.
• Commercial actors misaligned with health can cause or exploit this destabilization.
• Destabilized governance is relevant to commercial determinants of health research.
• Destabilized governance warrants more attention in health and climate policymaking.
• Public policy responses should prioritize commercial and wider determinants of health.

## 1 Introduction

As 2025 saw a battle between internationalism, diplomacy and sustainable development, and their headwinds of trade protectionism, cultural exceptionalism and geopolitical rivalry, it is easy to forget that climate change continues to rip, unabating, at the seams of public policy institutions. Short-term political crises, whether unplanned or self-fulfilling, can overshadow efforts in long-term, multisectoral governance for human health and planetary flourishing. Every moment, ‘anthropogenic’ climate change is arising from billions of exchanges in fossil fuel energy. But despite the ‘anthropo-’ prefix, these exchanges are not isolated between humans. Rather, climate change is fuelled by ‘commercio'-genic functions, including industrialization, globalization, economic interdependence and trade liberalization. Together with political norms that proxy gross domestic product (GDP) as economic development, these functions incentivize the unprecedented consumption of fossil fuels.

Perspectives on commerciogenic functions vary. Policymakers and economists question how to decarbonize while mitigating against the risks of a disordered transition [[1], [2], [3], [4], [5]]. While eco-modernists argue we can decouple economic development from environmental deterioration, through technological innovations and energy efficiencies [6], eco-Marxists reject hoping for technological fixes from a rampantly-emitting status-quo, and urgently call for precautions against a ‘fossil capitalism capitalocene’ [7]. New materialists, in turn, describe commerciogenic functions within an ‘uncanny Anthropocene’, unpredictably recoiling against damage done by humans, dispassionately and for perceived necessity [8]. Political scientists argue commerciogenic functions influence the governance of a ‘polycrisis’, where climate change deteriorates the conditions for policymaking by intersecting with crises in geopolitics, global health and human rights [[9], [10], [11], [12]]. All meanwhile, debates continue about the yet-to-be-realized emissions reductions by post-industrial, high-GDP economies needed to avoid global climate tipping points [[13], [14], [15], [16]].

Though disciplinary perspectives vary, two similarities persist. First, commerciogenic functions are rarely discussed with the language of public health. This matters because governing these functions is messy for health and health equity. The relationship between climate change and human development is non-linear [17]: consuming fossil fuels has advanced quality of life and livelihoods for many, but at an unequal pace. Yet, climate change inequitably exposes all of us to excess and avoidable diseases and deaths, degraded environments and, based on current trajectories, unfair and unchartered waters of a ‘worse’ yet to come. The World Health Organization (WHO) has described climate change as the *“…single biggest health threat facing humanity”* [18]. The physical and mental health and health equity impacts of the triple planetary crisis of climate change, biodiversity loss and pollution are well-established [[19], [20], [21], [22], [23], [24]]. In response, normative climate and health policy and practice is emerging. Particular attention has been given to climate-resilient and decarbonized health sectors [25], responding to extreme weather events as health emergencies [26], and developing high-quality data and indicators [[27], [28], [29], [30]].

Second, businesses do not passively exist within commerciogenic functions, but actively influence them to externalize their harms and maximize their perceived incentives. Clearly, decarbonized health services and health emergency responses are important entry points for climate and health policy and practice. However, they risk reperforming downstream and biomedical paradigms: trapping the ‘climate and health’ discourse within clinics and hospitals. Indeed, the two indicators evaluating WHO’s strategic objective in its Fourteenth General Programme of Work 2025–2028, to *“…respond to climate change [as] an escalating health threat”,* are measuring healthcare sector greenhouse gas emissions, and national climate change and health capacity plans, respectively [31]. Instead, the health impacts of climate change are also ‘determined’ by the structures, systems and conditions in which we live. Specifically, the impacts of commercial actors, their activities, their enabling economic systems, and their wider norms on climate change are examples of ‘commercial determinants of health’ [32]. Examples of commercial determinants of health include that:

• Commercial actors destroy biodiversity, pollute, and degrade ecology through the manufacture, distribution, sale and wastage of their products and services [33].
• Over 70 % of major post-industrial revolution CO_2_ emissions can be traced to just 78 corporate and state producing entities [34].
• Decades of industrial disasters have exposed the living world and its inhabitants to toxic hazards, accidents, waste and water dumping, and illegal deforestation [[35], [36], [37]].
• Annual global waste generation is expected to increase from 2.01 billion tonnes in 2016 to 3.4 billion tonnes by 2046 [38].
• Agriculture from just seven commodities (cattle, oil palm, soy, cocoa, plantation rubber, coffee, plantation wood fibre) was attributed to 71.9 million hectares of deforestation between 2001 and 2015 [39].
• Fossil fuel, plastics, and agrichemical industry actors have used denial, delay and obstruction strategies to obfuscate decades of climate policymaking [[40], [41], [42], [43], [44]].

This perspective explores two questions. First, how does climate change have intermediary destabilizing impacts on systems of governance necessary for health and health equity? Second, how might commercial actors misaligned with health cause, worsen or exploit conditions of destabilized governance? Reflecting on these impacts, we discuss ways to advance and integrate health and climate policy and practice, with particular attention to the commercial determinants of health.

Following precedent for investigating emerging commercial determinants of health, this perspective combines searches of PubMed, Google Scholar and the internet to identify relevant evidence from academic and grey literature, and media sources [45]. It is also informed by the corresponding author’s prior experiences working on social determinants of health policy for an intergovernmental agency. Acknowledging conceptual complexity around ‘health governance’, we focus on ‘governance for health and health equity’, defined as the pursuit of health through whole-of-government and whole-of-society actions [46]. This governance is both ‘networked’ (governing institutional relationships, structures and norms) and an ‘ideal’ (optimising ‘good governance’ decisions within these institutions, structures and norms) [47]. We describe impacts using a ‘PEST’ analysis (of political, economic, social, and technological factors): a simple but widely-used tool to assess macroenvironmental conditions and understand strategic risk [48]. We also focus on literature describing how practices of commercial actors interact with systems of governance as determinants of health. Commercial practices matter because health is not only determined by consuming a product or service, but also by corporate influence through political, financial, labour, supply chain, scientific, marketing and reputational management activities [32]. Reciprocally, climate change determines how commercial actors operate, enabling industries who exploit destabilized systems in a manner misaligned with health, while threatening the viability of others coherent with health.

## 2 How climate change is destabilizing systems of governance and making them vulnerable to commercial exploitation – a concern for health and equity

Climate change is destabilizing the foundational systems of politics, economics, social function and technological advancement that govern our societies. This destabilization has widespread and unpredictable consequences for health and health equity, and is caused, worsened and/or exploited by several commercial actors misaligned with health. Using the PEST format, these changes are summarized in the following paragraphs, with examples and references provided in Table 1.

**Table 1:** A ‘PEST’ analysis of risks to health and health equity from the destabilization by climate change to systems of governance, and potential misalignments between commercial and health interests.

Systems	System destabilization from climate change with consequence for health	Examples of potential misalignment between commercial and health interests
**Political systems destabilization**	Climate change demands the diversion of public sector capacity away from health policy functions, and towards climate mitigation, adaptation and response functions [50]	• Corporate capture over forming constitutions, laws and policies, their implementation, and related governmental and nongovernmental (legal, civil society, media) accountability mechanisms [51]. • The displacement of state competencies for regulatory climate policy, by neoliberal and market-directed mechanisms [52]. • The disempowering of the modern nation state to set commercial and economic conditions, instead being participants in a global, capitalized economy erratically co-produced between states and economic actors [53]. • A resistance of investor-owned fossil energy companies to being scrutinized under the principle of ‘common but differentiated responsibilities’ [49].
	Governments allow a growing gap between the financing needed for climate mitigation and adaptation, and what they are willing to spend [55].	• The risk of normalising public policy objectives as only achievable with conditions-based private sector finance, where de-risked private finance is not risk-shared, but re-risked onto governments and institutions [56], or normalising market-aligned development financing such as loans, non-grant instruments, trade liberalization or privatization [57,58]. • Risk of entrenching market-led approaches to climate policy that perpetuate externalities and offer an incomplete economic assessment of benefits and harms [59].
	Declining public trust in policymakers to respond effectively to climate change leads to political instability and power changes within and between nations [50], and the popularization of ideologies and governance approaches less associated with evidence-based health policymaking [61,62].	• The proliferation of climate and health mis- and disinformation on social media [64] or by distorting scientific credibility [106]. • The risk of recasting ‘hard’ mandatory commercial obligations to human rights as ‘soft’ corporate responsibilities to respect human rights [63]. • Commercial actors undermining solidaristic and collective approaches to climate change adaptation, by positioning risk as individualized, technical and calculative [116]. • Presenting commercial actors as trustworthy alternatives to governments, despite different modus operandi and incentive structures [117].
	Climate change increases the frequency and severity of health emergencies.	• Political underestimation of how companies who make essential medicines and health technologies will act in self-interest, while overestimating hopes for these companies to act in solidarity with health, as experienced with the COVID-19 Vaccine Global Access Facility (COVAX) [65]. • Symbiosis in corporate and political positioning to deny technology transfer clauses in health- and climate-related negotiations [58]. • The strategic orientation of Corporate Social Responsibility (CSR) in a health emergency to obtain future tangible benefits, such as increased sales and financing conditions, and intangible benefits, such as political goodwill and reputation management [54]. • The privatization of health-essential services, such as private firefighting industries, dividing public ‘rights’ and private ‘wants’ [60].
**Economic systems destabilization**	Climate change dismantles fiscal norms, resulting in existential losses to GDP [118], and reducing budgetary capacity and disposable income for spending to enable health.	• Catastrophic and unquantifiable impacts of climate change to the global economy, unpredictably affecting the function of governments, commercial actors, and economic norms at large. • The commercial recasting of economic systems that have accelerated climate change to justify new opportunities in climate adaptation and mitigation for neoliberal market expansion and value extraction, such as energy carbon markets [51]. • Companies reversing their climate commitments on perceived economic grounds [86].
	Climate change reshapes employment, labour and wage market structures, resulting in economic insecurity as a negative determinant of health [50,[68], [69], [70]].	• Increased casualization of labour leading to poorer general health, mental health, emotional and workplace wellbeing, and labour force participation [77,78]. • Loss of medical coverage, illness pay and workplace health adjustments as employment health and social welfare protections, all resulting in increased vulnerability to catastrophic out-of-pocket medical expenditure [83,84]. • The consequences of digitalization for the economic security of those employed in supply chains. • Employer pressures on economically-insecure employees to work longer hours, show presenteeism, and work within disorganized occupational health and safety systems [84]. • Modern slavery and informal economy violations of occupational health and human rights [[79], [80], [81]]. • Disruption by climate change to natural-resource-based livelihoods drives people towards health-harming commercial activities, such as illicit drug production [82].
	Climate change disrupts supply chains, inflationary pressures [71], resource prices and resource availability [72], reducing access to nutritious foods [[73], [74], [75]] and essential medicines and health technologies [66,67].	• Justifying the proliferation of processed foods and beverages as necessary for ‘predictable’ supply chains, trading off supposed protection against environmental variability with an accelerated transition to energy-dense, nutrient-poor food systems [57,73]. • Demand surges for medicines and health technologies leading to price fluctuations, unequal distribution and access, and other market failures [91]. • Potential for exceptional and concentrated commercial profitability at a time of rising prices, with inequities across food commodity markets [90]. • Crop yield volatility and food price inflation resulting in hunger and malnutrition [119]. • Exploiting health-essential products for commercial gain, such as armed groups extorting scarce essential medical supplies [87,88].
	Countries respond to climate change weakening their national economic stability by adopting climate-harming, growth-centric responses, that ‘double-down’ on perverse market failures incompatible with health.	• The ongoing enablement of fossil fuel subsidies, despite clear evidence of their economic inefficiencies and harms to climate and health [[120], [121], [122]]. • Industrial symbioses that ‘lock-in’ unsustainable material systems dependent on fossil fuel extraction [33] • Trade-offs for the viability of commercial operations with government interests that may undermine the attainment of low-carbon transition policies, such as state-owned petrochemical companies [89]. • Regulatory chill or policy reversal in response to perceived economic difficulties, such as relaxing vehicle emissions rules [123].
	Climate change renders health-protecting industries unviable.	• Threat of market withdrawal or non-viability of the health and home insurance industries [85]. • Changing norms about whether insurance can adequately compensate for the harms of climate change, risking sector failure and government bailouts [124]. • Threat of commercial abandonment from businesses relied on to sustain health-enabling resources, such as businesses transforming otherwise unproductive land for food production [92,93], leading to food insecurity if operations cease. • Loss of economic security for those employed in the supply chains of such industries.
**Social systems destabilization**	Climate change increases the frequency and severity of social and civil unrest [50].	• Civil unrest as a risk to businesses and their insurers, endangering the safety of employees and customers while interrupting business continuity and assets [125]. • Financial incentives for private military and security companies to create and respond to civil unrest [99]. • As previously discussed, use of digital media to proliferate mis- and disinformation.
	Climate change increases the frequency and severity of conflict and violence [50].	• Risks of violence resulting from the impact of commercial practices on local communities, including diminished access to water supplies from water diversion, irresponsible waste and pollution management, and food scarcity [45]. • As previously discussed, the range of commercial practices to exploit conditions of social unrest in a manner misaligned with health.
	Climate change accelerates disease spread [50].	• Structural dependencies on market-based solutions to develop a needed medicine or health technology, and inequities in price and access strategies [65]. • Commercial actors may subject their workforce to heightened risk of disease infection and transmission in pursuit of maximal production [76]. • As discussed per GDP, a threat to economic functioning at large through the emergence of an unpredictable pandemic.
	Climate change has unpredictable and catastrophic impacts on forced human migration [96].	• Commercial exploitation risks to health in migration arise from human and sex trafficking, drug addiction, destruction or expropriation of property, and violence in all forms by organized crime and other nefarious commercial actors [97,98]. • The delegation of border control sovereignty to commercial actors, risking lack of oversight and accountability, and incentive structures to violate international humanitarian law [100,101]. • Vagueness over how legal norms for regulating armed forces conduct in migration contexts are applied to the private security industry [102]. • Poor quality health conditions in privatized immigrant detention, housing, welfare and administrative services for asylum seekers or refugees [[103], [104], [105]]. • A disproportionate risk to catastrophic health care expenditure among migrant workers arising from a lack of access to health services [94,95].
**Technological systems destabilization**	Climate change and biodiversity loss leads to extinction and scarcity of organic compounds that undermines new drug discovery [19].	• Increased difficulty for new medical discoveries, creating a potential disincentive for pharmaceutical research and development in areas of public health need [126].
	Climate change disrupts the ‘traditional technologies’ of Indigenous biotic resources [19]	• Risk of the commercial co-option of community-owned Indigenous knowledge, such as patents on traditional nutritious crops or the promotion of genetic research [114]. • Ongoing extractive industry destruction of Indigenous land and resources [115]. • Normalization of an inevitability that Indigenous knowledge and subsistence continue to be lost to extractive expansion as acceptable industrial policy, while weakening civil society opposition [113]. • The displacement of the overall impacts of these industrial projects on Indigenous livelihoods by corporate-led initiatives to integrate Indigenous persons and their knowledge into industrial projects once established [127].
	Climate events cause power outages or network disruptions, impacting health care services [108,109].	• Commercial actors can exacerbate poorer health outcomes during a network disruption, such as exposing people ton carbon monoxide poisoning, or enabling heightened risks of gun violence, [107]. • Trade-offs in energy and infrastructure demands of competing digital technologies [111].
	Climate change forces uncomfortable trade-offs about demand for resources needed for both human life and economic growth.	• Examples include trade-offs over water needed for both sustenance and food security but also cooling data centres [112] • Commercial agriculture trading off food security and employment for land dispossession, lost hunting and foraging [110].

### 2.1 Political systems

By placing additional and unpredictable demands on public services and population health needs, climate change is destabilising the political fabric of all governments, regardless of scale or governance context. The longer insufficient political action to address climate change continues, the greater the ultimate impact of mitigation, adaptation and response efforts on diminishing public sector capacities for policymaking, diverting precious resources away from health and health-related policy competencies [[49], [50], [51], [52], [53]]. Doing so creates opportunities for corporate capture over health and wider public policy functions [[54], [55], [56], [57], [58], [59], [60]]. It also has more diffuse impacts for corporate power over public governance. These include the role of social media in declining public trust that politicians can respond effectively to climate change and its health impacts, pushing people to modes of political organization misaligned with health [50,[61], [62], [63], [64]], or subsuming the nation state within global systems of capital that are increasingly directed by market actors [53,59], such as in response to health essential medicines and services [58,60] and climate-related health emergencies [54,65].

### 2.2 Economic systems

Climate change is likely to lead to foundational and unpredictable impacts on macroeconomic performance indicators such as GDP, employment rates, job security, inflation and resource scarcity [50,[66], [67], [68], [69], [70], [71], [72], [73], [74], [75]]. These impacts undermine economic security by restructuring labour and wage markets with consequences for economic security [50,[68], [69], [70]] and occupational health [[76], [77], [78], [79], [80], [81], [82]], putting pressure on the costs of accessing health-essential products and services [83,84], undermining budgetary allocations for health [50], and forcing uncomfortable trade-offs between chasing growth-, activity-based and planet-harming economic performance that runs contrary to the economic reforms needed to better value health, planet and livelihoods [57,73,82,[85], [86], [87], [88], [89]]. Commercial actors responsible for exacerbating climate change are also seeing new opportunities to recast existing systems that have harmed health and health equity as fixable, through market-led mechanisms such as neoliberal expansionism, unproven technologies, the innovation paradigm and in creating new means of value extraction [51,90,91], while commercial sectors that can benefit health face threats of market non-viability from climate change [85,92,93].

### 2.3 Social systems

Climate change does not only impact on health in social systems through a heightened risk of disease transmission [50], but also from growing climate-caused civil unrest, conflict, violence and forced human migration [45,[94], [95], [96]]. In inflamed social contexts, commercial actors misaligned with health can weaponize discontent and subjugate vulnerable populations. This includes through the proliferation of private security, illicit drug and human trafficking operations [50,[97], [98], [99]], suppressing unrest at the privatization of community resources [45], or the privatization of sovereign functions such as border security [100,101], military force [102] or services for migrant populations [[103], [104], [105]]. Social attitudes to health and climate governance are also reshaped through the proliferation of mis- and disinformation in digital media [64,106].

### 2.4 Technological systems

Last, climate change is undermining the development and optimization of technologies relied upon to improve health. Examples include undermining access to organic compounds necessary for medical research and development [19], or disruptions to the power and digital infrastructure needed to sustain health care services and medical supply chains [[107], [108], [109]]. Technological demands are also creating uncomfortable trade-offs over resources needed both for human life and economic growth [[110], [111], [112]]. Importantly, technological risks include the risks of climate change to Indigenous technologies, and the risks of the commercial co-option of these technologies in a manner that further disenfranchizes Indigenous communities from ownership of their knowledge [[113], [114], [115]].

## 3 Actions for advancing and integrating climate and health policies

Effective governance for health and health equity depends on stable political, environmental, social and technological systems. Because of past inaction, this stability is now at the mercy of climate change. Both the destabilising impacts of climate change on governance, and the resulting policy responses from governing bodies, create path dependencies that shape our collective livelihoods and the future of the planet [128]. Governance for health and health equity is currently undermined by commerciogenic functions that force incoherent trade-offs between planetary viability, market-driven development, and safeguarding human rights, including the right to health [129]. We propose the impacts of climate change on systems of governance for health and health equity deserve far greater attention in policy and practice, as do the ways that commercial actors can cause, worsen and exploit destabilized governance in a manner misaligned with health.

Four actions to advance and integrate climate and health policies are recommended:

First, governments should cooperate to upweight and mainstream attention to the determinants of health and health equity in intersectoral and multisectoral health and climate policies and plans, within and across governance contexts, from local to global levels. This does not mean neglecting existing actions to create climate-resilient health sectors, decarbonize health services, respond to climate health emergencies, or build knowledge on disease pathways and burdens. Each of these improves the case for comprehensive action on ‘climate and health’. Rather, it means health and climate policymakers must recognize that without cooperating to tackle ‘supply-side’ drivers of climate change, governments will be trapped with ever-worsening ‘demand-side’ burdens on their health systems, undermining the efficacy of public policy at large.

Second, governments should prioritize responding to commercial activities as determinants of climate change and health in policy and planning. This perspective highlights multiple ways that commercial actors cause, worsen and exploit destabilized governance in a manner misaligned with health. Resulting policies must not only address health-harming products and services, but also safeguard against a range of commercial practices that influence public and political discourse and downplay corporate impacts on climate change. One precedent is the WHO Framework Convention on Tobacco Control (WHO FCTC), which describes and regulates a wide range of commercial practices undermining public health. Attending to commercial impacts requires going beyond political commitments, to understand how commitments are implemented. It also creates opportunities to build new multisectoral constituencies of support in policy capacities outside of public health, but where health is co-beneficial. Examples include health aligning with voices working to: safeguard the public interest from corporate capture; address violence and commercial exploitation as a human security issue; advance wellbeing-based economic approaches; and/or address climate change as an existential threat to business.

Third, governments and international finance institutions (IFIs) should acknowledge their past approaches have accelerated the changing climate that now existentially threatens sustainable economic development. Learning from this, they should urgently re-value their approaches to market externalities, climate financing and human and institutional capital development. Necessary normative policy shifts include: treating human health and planetary flourishing as foundational prerequisites for sustainable economic and human development; urgently mobilising domestic and international financing for effective, cost-effective and equitable health and climate interventions; and championing and scaling new industrial policy ecosystems aligned with health and the public interest. It also means unapologetically rejecting the proliferation of commercial activities and consumptive systems misaligned with health and climate change, and disputing commercial opportunism to present market externalities from climate change as appropriate or inevitable.

Fourth, as custodians of sustainable development and human rights, governments and civil society have opportunities to mainstream coherent policies that champion resilient economies, sustainable development, human health and planetary flourishing. Many health-harming industries leverage business models that accelerate climate change while causing death and disease. Yet, health, planetary, and economic policy coherence is possible, and should be celebrated. We have many examples. Because healthier societies have an increased adaptative capacity, and are inherently more resilient to climate shocks, health investments can maximize climate adaptation and resilience efforts [130,131]. Integrating climate-sensitive components into domestic and international financing mechanisms for health has been underutilized [[132], [133], [134]]. And universal owners – holders of highly-diversified and long-term financial portfolios across global markets – share interests in climate mitigation and adaptation, albeit motivated by avoiding capital risks [135]. Indeed, caution should be exercised against making overgeneralized binaries of ‘pro-health and climate change’ as ‘anti-profit or economic development’: health and climate resilience are foundational to the economic systems we desperately need, not incompatible from them.

## 4 Conclusion

Climate change is destabilizing political, economic, social and technological systems of governance that are crucial for health and health equity. This destabilization is happening in fundamental, urgent and unpredictable ways, with transformative implications for societies, economies and the function of public policy. All are integral to health and health equity outcomes. The impacts of climate change on destabilized conditions of governance for health and health equity are also caused, worsened and exploited by commercial actors misaligned with health, making this a key area of inquiry for commercial determinants of health policy and practice. Recommendations for intersectoral and multisectoral health and climate policy are offered. We hope this perspective is a timely and thought-provoking synthesis of existing thinking. It would be a pleasure to further develop this thinking – including with those working across disciplines, who may only now be considering public health as an important component of climate governance.

## CRediT authorship contribution statement

**Daniel Hunt:** Writing – review & editing, Writing – original draft, Project administration, Methodology, Investigation, Formal analysis, Conceptualization. **Britta K Matthes:** Writing – review & editing, Supervision, Project administration, Investigation.

## Declaration of competing interest

The authors declare the following financial interests/personal relationships which may be considered as potential competing interests:

Daniel Hunt reports having undertaken consulting or advisory relationships with the World Health Organization, Clean Air Fund and the NCD Alliance.

Britta K Matthes reports her research post being funded by Bloomberg Philanthropies as part of the Bloomberg Initiative to Reduce Tobacco Use.

## References

[1] Di Febo E., Angelini E. (2025). Transition Risk in Climate Change: a literature review. Risks.

[2] Lamperti F., Roventini A. (2022). Beyond climate economics orthodoxy: impacts and policies in the agent-based integrated-assessment DSK model*. Eur J Econ Econ Polic.

[3] Fried S., Novan K., Peterman W.B. (2022). Climate policy transition risk and the macroeconomy. Eur Econ Rev.

[4] Nelson J.A. (2008). Economists, value judgments, and climate change: a view from feminist economics. Ecol Econ.

[5] Green F. Transition policy for climate change mitigation: who, what, why and how. Canberra, Australia: Australian National University; 2018. Working Paper 1807. Accessed 14 May 2024. Available from: https://climatestrategies.org/wp-content/uploads/2019/09/Transition-Policy_Green_2018_FINAL.pdf.

[6] Sconfienza U.M. (2020). Incomplete Ecol Futures World Futures.

[7] Malm A. (2016).

[8] Ejsing M. (2023). The arrival of the Anthropocene in social theory: from modernism and Marxism towards a new materialism. Sociol Rev.

[9] Helleiner E. (2024). Economic Globalization’s polycrisis. Int Stud Q.

[10] Lawrence M., Homer-Dixon T., Janzwood S., Rockstöm J., Renn O., Donges J.F. (2024). Global polycrisis: the causal mechanisms of crisis entanglement. Glob Sustain.

[11] Krogmann D. (2025). Liberal environmentalism and climate change in the polycrisis. Glob Sustain.

[12] Jayasuriya K. (2023). Polycrisis or crises of capitalist social reproduction. GSCJ.

[13] Kelly O., Thombs R.P., Jorgenson A. (2021). The unsustainable State: greenhouse gas emissions, inequality, and Human well-being in the United States, 1913 to 2017. Socius.

[14] Garrity E.J. (2012). Tragedy of the commons, business growth and the fundamental sustainability problem. Sustainability.

[15] Bjørnskov C. (2024). Economic freedom and the greenhouse gas Kuznets curve. Eur J Polit Econ.

[16] Jakob M., Lamb W.F., Steckel J.C., Flachsland C., Edenhofer O. (2020). Understanding different perspectives on economic growth and climate policy. WIREs Clim Change.

[17] Nathwani J., Lind N., Renn O., Schellnhuber H.J. (2021). Balancing health, economy and climate risk in a multi-crisis. Energies.

[18] World Health Organization. Climate change and noncommunicable diseases: connections, https://www.who.int/news/item/02-11-2023-climate-change-and-noncommunicable-diseases-connections; 2023 [accessed 14 April 2025].

[19] Díaz S., Settele J., Brondízio E.S., Ngo H.T., Guèze M., Agard J. (2019).

[20] Kjellstrom T., Butler A.J., Lucas R.M., Bonita R. (2010). Public health impact of global heating due to climate change: potential effects on chronic non-communicable diseases. Int J Public Health.

[21] Sharma A.K., Sharma M., Sharma A.K., Sharma M., Sharma M. (2023). Mapping the impact of environmental pollutants on human health and environment: a systematic review and meta-analysis. J Geochem Explor.

[22] Shetty S.S., D D., S H., Sonkusare S., Naik P.B., N S.K. (2023). Environmental pollutants and their effects on human health. Heliyon.

[23] (2024). World Health Organization. A77/A/CONF./7. Clim change health.

[24] World Health Organization. COP29 special report on climate change and health: health is the argument for climate action. World Health Organization; 2024.

[25] Hough E., Cohen Tanugi-Carresse A. (2024). Supporting decarbonization of health systems-A review of international policy and practice on health care and climate change. Curr Env Health Rep.

[26] Ebi K.L., Vanos J., Baldwin J.W., Bell J.E., Hondula D.M., Errett N.A. (2021). Extreme weather and Climate change: population health and health system implications. Annu Rev Public Health.

[27] Li A., Toll M., Bentley R. (2023). Mapping social vulnerability indicators to understand the health impacts of climate change: a scoping review. Lancet Planet Health.

[28] Liu A.Y., Trtanj J.M., Lipp E.K., Balbus J.M. (2021). Toward an integrated system of climate change and human health indicators: a conceptual framework. Clim Change.

[29] Palmeiro-Silva Y., Aravena-Contreras R., Izcue Gana J., González Tapia R, Kelman I. (2024). Climate-related health impact indicators for public health surveillance in a changing climate: a systematic review and local suitability analysis. Lancet Reg Health Am.

[30] Sena A., Ebi K.L., Freitas C., Corvalan C., Barcellos C. (2017). Indicators to measure risk of disaster associated with drought: implications for the health sector. PLoS One.

[31] World Health Organization. A global health strategy for 2025-2028 - advancing equity and resilience in a turbulent world: fourteenth general programme of work. Geneva, Switzerland: World Health Organization; 2025.

[32] Gilmore A.B., Fabbri A., Baum F., Bertscher A., Bondy K., Chang H.-J. (2023). Defining and conceptualising the commercial determinants of health. Lancet.

[33] Velenturf A.P.M., Purnell P. (2021). Principles for a sustainable circular economy. Sustain Prod Consum.

[34] InfluenceMap. The Carbon Majors Database, https://influencemap.org/pressrelease/Carbon-Majors-57-fossil-fuel-and-cement-producers-linked-to-80-of-global-fossil-CO2-emissions-since-the-Paris-Agreement-27590 2024; 2024 [accessed 19 February 2025].

[35] Cvetković V.M., Renner R., Jakovljević V. (2024). Industrial disasters and hazards: from causes to consequences—A holistic approach to resilience. Int J Disaster Risk Manag.

[36] Council on Foreign Relations. Timeline: 1912 -2020 ecological disasters, https://www.cfr.org/timeline/ecological-disasters; 2025 [accessed 27 May 2025].

[37] Johnston J., Cushing L. (2020). Chemical exposures, health and environmental justice in communities living on the fenceline of industry. Curr Env Health Rep.

[38] World Bank. Global waste to grow by 70 percent by 2050 unless urgent action is taken: world Bank Report, https://www.worldbank.org/en/news/press-release/2018/09/20/global-waste-to-grow-by-70-percent-by-2050-unless-urgent-action-is-taken-world-bank-report; 2018 [accessed 19 February 2025].

[39] Goldman E.D., Weisse M., Harris N., Schneider T. Estimating the role of seven commodities in agriculture-linked deforestation: oil palm, soy, cattle, wood fiber, cocoa, coffee, and rubber. Washington DC, United States of America: World Resources Institute; 2020. Accessed 19 February 2025. Available from: https://www.wri.org/research/estimating-role-seven-commodities-agriculture-linked-deforestation-oil-palm-soy-cattle.

[40] Pulver S. (2017). A politics of the public sphere: eNGOs and oil companies in the international climate negotiations, 1987–2001. Gov Extr Ind.

[41] Ralston R., Carlini G., Johns P., Lencucha R., Radvany R., Shah D. (2023). Corporate interests and the UN treaty on plastic pollution: neglecting lessons from the WHO Framework Convention on Tobacco Control. Lancet.

[42] Kinol A., Si Y., Kinol J., Stephens J.C. (2025). Networks of climate obstruction: discourses of denial and delay in US fossil energy, plastic, and agrichemical industries. PLOS Clim.

[43] Brulle R.J. (2023). Advocating inaction: a historical analysis of the Global Climate Coalition. Env Polit.

[44] Franta B. (2022). Weaponizing economics: big oil, economic consultants, and climate policy delay. Env Polit.

[45] Bellis M.A., McManus S., Hughes K., Adisa O., Ford K. (2024). The commercial determinants of violence: identifying opportunities for violence prevention through a public health-based framework analysis. Int J Env Res Public Health.

[46] Kickbusch I., Gleicher D. Governance for health in the 21st century. World Health Organization. Regional Office for Europe; 2012.

[47] Barbazza E., Tello J.E. (2014). A review of health governance: definitions, dimensions and tools to govern. Health Policy (N Y).

[48] Sammut-Bonnici T., Galea D. (2015). Wiley encyclopedia of management.

[49] Frumhoff P.C., Heede R., Oreskes N. (2015). The climate responsibilities of industrial carbon producers. Clim Change.

[50] Sellers S., Ebi K.L., Hess J. (2019). Climate Change, Human health, and Social stability: addressing interlinkages. Env Health Perspect.

[51] Fletcher R. (2012). Capitalizing on chaos: climate change and disaster capitalism. Ephemera: Theory Polit Organ.

[52] MacNeil R., Paterson M. (2012). Neoliberal climate policy: from market fetishism to the developmental state. Env Polit.

[53] Danielsen D., Stark B. (2015). International law and its discontents: confronting crises.

[54] García-Sánchez I.-M., García-Sánchez A. (2020). Corporate Social responsibility during COVID-19 Pandemic. J Open Innov: Technol Mark Complex.

[55] UNFCCC (2024). COP29 UN Climate Conference Agrees to Triple Finance to Developing Countries, Protecting Lives and Livelihoods | UNFCCC.

[56] Mazzucato M. Financing the Sustainable Development Goals through mission-oriented development banks. New York, United States of America: UN Department of Economic and Social Affairs and UCL Institute for Innovation and Public Purpose; 2023. UN DESA Policy Brief Special Issue. Accessed 9 April 2025. Available from: https://www.ucl.ac.uk/bartlett/public-purpose/publications/2023/sep/financing-sustainable-development-goals-through-mission-oriented-development-0.

[57] World Health Organization. Economic and commercial determinants of health in small island developing states: noncommunicable diseases, mental health conditions, injuries and violence. World Health Organization; 2025.

[58] Muchhala B. (2022). The structural power of the State-finance nexus: systemic delinking for the right to development. Dev.

[59] Stilwell F. (2020). Marketising the environment. J Aust Polit Econ.

[60] Specht D. The rise of firefighters-for-hire exposes the inequality of climate-driven disasters. The Conversation, http://theconversation.com/the-rise-of-firefighters-for-hire-exposes-the-inequality-of-climate-driven-disasters-247851; 2025 [accessed 20 February 2025].

[61] Krange O., Kaltenborn B.P., Hultman M. (2021). Don’t confuse me with facts”—How right wing populism affects trust in agencies advocating anthropogenic climate change as a reality. Humanit Soc Sci Commun.

[62] Yan P., Schroeder R., Stier S. (2022). Is there a link between climate change scepticism and populism? An analysis of web tracking and survey data from Europe and the US. Inf Commun Soc.

[63] Ishikawa T. (2022). Corporate environmental responsibility in investor-state dispute settlement: the unexhausted potential of current mechanisms.

[64] Piatek S.J., Haines A., Larson H.J. (2024). We need to tackle the growing threat of mis- and disinformation about climate change and health. BMJ.

[65] itad. Final report: COVAX Facility and AMC Formative review and Baseline Study. Geneva, Switzerland. GAVI, The Vaccine Alliance; 2023. Accessed 14 May 2025. Available from https://www.itad.com/project/evaluating-the-effectiveness-of-covid-19-vaccine-delivery-by-the-covax-initiative/.

[66] Ahlqvist V., Dube N., Jahre M., Lee J.S., Melaku T., Moe A.F. (2022). Supply chain risk management strategies in normal and abnormal times: policymakers’ role in reducing generic medicine shortages. Int J Phys Distrib Logist Manag.

[67] Kaplan W.A., Hamer D.H., Shioda K. (2025). The potential impact of climate change on medication access and quality deserves far more attention. One Health.

[68] Begum A., Hamid S.A. (2021). Impoverishment impact of out-of-pocket payments for healthcare in rural Bangladesh: do the regions facing different climate change risks matter?. PLoS One.

[69] International Labour Organization. The employment impact of climate change adaptation. input document for the G20 climate sustainability working group. Geneva, Switzerland: 2018.

[70] Liu T.-Y., Lin Y. (2023). Does global warming affect unemployment? International evidence. Econ Anal Policy.

[71] Kotz M., Kuik F., Lis E., Nickel C. (2024). Global warming and heat extremes to enhance inflationary pressures. Commun Earth Env.

[72] Haile M.G., Wossen T., Tesfaye K., von Braun J (2017). Impact of climate change, weather extremes, and price risk on global food supply. EconDisCliCha.

[73] Davis K.F., Downs S., Gephart J.A. (2021). Towards food supply chain resilience to environmental shocks. Nat Food.

[74] Ghadge A., Wurtmann H., Seuring S. (2020). Managing climate change risks in global supply chains: a review and research agenda. Int J Prod Res.

[75] IPCC (2022).

[76] Freeman T., Baum F., Musolino C., Flavel J., McKee M., Chi C. (2023). Illustrating the impact of commercial determinants of health on the global COVID-19 pandemic: thematic analysis of 16 country case studies. Health Policy.

[77] Jaydarifard S., Smith S.S., Mann D., Rossa K.R., Nikooharf Salehi E, Gnani Srinivasan A (2023). Precarious employment and associated health and social consequences; a systematic review. Aust N Z J Public Health.

[78] Jiménez M. (2021). https://desapublications.un.org/policy-briefs/undesa-policy-brief-95-what-triggers-economic-insecurity-and-who-most-risk.

[79] Brown D., Boyd D.S., Brickell K., Ives C.D., Natarajan N., Parsons L. (2021). Modern slavery, environmental degradation and climate change: fisheries, field, forests and factories. Environ Plan E: Nat Space.

[80] Sparks J.L.D., Boyd D.S., Jackson B., Ives C.D., Bales K. (2021). Growing evidence of the interconnections between modern slavery, environmental degradation, and climate change. One Earth.

[81] Wang Y., Lotfi M. (2024). How climate change and modern slavery interact in the supply chain: a conceptual model development through a systemic review. Bus Ethics Environ Responsib.

[82] Lucas B. (2024).

[83] Benach J., Muntaner C., Solar O., Santana V., Quinlan M. (2010). Introduction to the WHO Commission on Social Determinants of Health Employment Conditions Network (EMCONET) study, with a glossary on employment relations. Int J Health v.

[84] Quinlan M. (2015).

[85] Herweijer C., Ranger N., Ward R.E.T. (2009). Adaptation to Climate change: threats and opportunities for the insurance industry. Geneva Pap Risk Insur Issues Pr.

[86] Igini M. (2025). These Co Are Aligning Trump’s Anti-Clim Agenda.

[87] Breslawski J. (2022). Armed groups and public health emergencies: a cross-country look at Armed groups’ Responses to Covid-19. J Glob Secur Stud.

[88] News. Haiti B.B.C. (2023). Where aid deliv depends talk 300 gangs.

[89] OECD. Climate change and low-carbon transition policies in state-owned enterprises. Paris, France: OECD; 2022. Accessed 1 February 2025. Available from: https://www.oecd.org/en/publications/climate-change-and-low-carbon-transition-policies-in-state-owned-enterprises_e3f7346c-en.html.

[90] Harvey F. Rec Profits Grain Firms Food Crisis Prompt Calls Windf Tax. https://www.theguardian.com/environment/2022/aug/23/record-profits-grain-firms-food-crisis-calls-windfall-tax; 2022 [accessed 15 May 2025].

[91] Engineering National Academies of Sciences, Health and Medicine Division, Board on Health Sciences Policy, Committee on Security of America’s Medical Product Supply Chain, Shore C., Brown L., et al. Causes and consequences of medical product supply chain failures. building resilience into the nation’s medical product supply chains, National Academies Press (US); 2022. 36070409

[92] Kosmas C., Kairis O., Karavitis C., Acikalin S., Alcalá M., Alfama P. (2015). An exploratory analysis of land abandonment drivers in areas prone to desertification. CATENA.

[93] Reed M.S., Stringer L.C. (2016).

[94] Ashour M., Abuzaid A., Korachais C. (2013). Catastrophic health expenditure and entitlement to health services in the occupied Palestinian territory: a retrospective analysis. Lancet.

[95] Liu L., Zhang X., Zhao L., Li N. (2019). Empirical analysis of the status and influencing factors of catastrophic health expenditure of migrant workers in western China. Int J Env Res Public Health.

[96] Schwerdtle P., Bowen K., McMichael C. (2017). The health impacts of climate-related migration. BMC Med.

[97] Becker S.O., Ferrara A. (2019). Consequences of forced migration: a survey of recent findings. Labour Econ.

[98] Sheu J.C., Torres M.I.M., Gordon M.R., Nguyen P.T., Coverdale J.H. (2021). Potential impact of climate change on Human trafficking: a narrative review. J Nerv Ment Dis.

[99] Martínez Hernández C.A., Camps-Febrer B. (2024). Securing sustainability: private military and security companies (PMSC) influence on european migration and climate po.

[100] Infantino F. (2021). State sovereignty, risk, and security when private companies practice border control. Secur Saf Era Glob Risks.

[101] McLean N. (2023). State sovereignty and private military and security companies in Australia. Aust J Polit Hist.

[102] Gammeltoft-Hansen T. (2015).

[103] Darling J. (2016). Privatising asylum: neoliberalisation, depoliticisation and the governance of forced migration. Trans Inst Br Geogr.

[104] Flynn M., Cannon C.J. (2009).

[105] Lethbridge J. (2017). http://www.world-psi.org/sites/default/files/documents/research/final_psi_epsu_psiru_privatisation_of_migration_and_refugee_services.pdf.

[106] Legg T., Hatchard J., Gilmore A.B. (2021). The science for profit Model—How and why corporations influence science and the use of science in policy and practice. PLOS ONE.

[107] Freese J., Richmand N.J., Silverman R.A., Braun J., Kaufman B.J., Clair J. (2006). Impact of citywide blackout on an Urban Emergency Medical Services system. Prehosp Disaster Med.

[108] Casey J.A., Fukurai M., Hernández D., Balsari S., Kiang M.V. (2020). Power outages and community health: a narrative review. Curr Env Health Rep.

[109] Kile J.C., Skowronski S., Miller M.D., Reissman S.G., Balaban V., Klomp R.W. (2005). Impact of 2003 power outages on public health and emergency response. Prehosp Disaster Med.

[110] Akinyi D.P., Ng’ang’a S.K., Girvetz E.H (2021). Trade-offs and synergies of climate change adaptation strategies among smallholder farmers in sub-Saharan Africa: a systematic review. Reg Sustain.

[111] Fritzsche K., Niehoff S., Beier G. (2018). Industry 4.0 and climate change—Exploring the science-policy gap. Sustainability.

[112] Andrews D., Flucker S., Whitehead B., Tozer R. (2017). Ashrae Winter Conference.

[113] Westman C.N., Joly T.L. (2019). Oil sands extraction in Alberta, Canada: a review of impacts and processes concerning indigenous peoples. Hum Ecol.

[114] Ratuva S. (2009). Commodifying cultural knowledge: corporatised western science and Pacific indigenous knowledge. Int Soc Sci J.

[115] Kennedy C.M., Fariss B., Oakleaf J.R., Garnett S.T., Fernández-Llamazares Á., Fa J.E. (2023). Indigenous Peoples’ lands are threatened by industrial development; conversion risk assessment reveals need to support Indigenous stewardship. One Earth.

[116] Lucas C.H., Booth K.I. (2020). Privatizing climate adaptation: how insurance weakens solidaristic and collective disaster recovery. WIREs Clim Change.

[117] Edelman. 2022 Special Report: trust and Climate Change. Chicago, United States of America: edelman; 2022. Accessed 20 February 2025. Available from: https://www.edelman.com/trust/2022-trust-barometer/special-report-trust-climate.

[118] Trust S., Saye L., Bettis O., Bedenham G., Hampshire O., Lenton T., et al. Planetary Solvency – finding our balance with nature. Global risk management for human prosperity. Exeter, United Kingdom: institute and Faculty of Actuaries, University of Exeter; 2025. Accessed 1 February 2025. Available from: https://actuaries.org.uk/news-and-media-releases/news-articles/2025/jan/16-jan-25-planetary-solvency-finding-our-balance-with-nature/.

[119] Horton H. (2024). Food insecurity rising in UK because of climate breakdown. Defra rep finds.

[120] Coady D., Parry I., Sears L., Shang B. (2017). How large are global fossil fuel subsidies?. World Dev.

[121] Rentschler J., Bazilian M. (2017). Reforming fossil fuel subsidies: drivers, barriers and the state of progress. Clim Policy.

[122] Skovgaard J., van Asselt H. (2019). The politics of fossil fuel subsidies and their reform: implications for climate change mitigation. WIREs Clim Change.

[123] Bloomberg UK.VW, Stellantis Are Biggest Win EU Relaxing CO2 Rules, https://www.bloomberg.com/news/articles/2025-03-05/vw-stellantis-can-exhale-as-eu-cuts-carmakers-some-slack-on-co2; 2025 [accessed 6 March 2025].

[124] Booth K., Martini G. (2024). Insurance in a changing climate: from commonsense to catastrophe. Finance Space.

[125] Allianz Commercial. Political violence and civil unrest trends 2025. London, United Kingdom: allianz Commercial; 2025. Accessed 14 April 2025. Available from: https://commercial.allianz.com/news-and-insights/reports/political-violence-and-civil-unrest-trends.html.

[126] Neergheen-Bhujun V., Awan A.T., Baran Y., Bunnefeld N., Chan K., dela Cruz T.E. (2017). Biodiversity, drug discovery, and the future of global health: introducing the biodiversity to biomedicine consortium, a call to action. J Glob Health.

[127] Stokes D.M., Marshall B.G., Veiga M.M. (2019). Indigenous participation in resource developments: is it a choice?. Extr Ind Soc.

[128] Vanhala L., Calliari E., Thomas A. (2023). Understanding the politics and governance of Climate change loss and damage. Glob Environ Polit.

[129] Sconfienza U.M. (2019). The post-sustainability trilemma. J Environ Policy Plan.

[130] Keim M.E. (2008). Building Human resilience: the role of public health preparedness and response As an adaptation to climate change. Am J Prev Med.

[131] Hess J.J., McDowell J.Z., Luber G. (2012). Integrating climate change adaptation into public health practice: using adaptive management to increase adaptive capacity and build resilience. Env Health Perspect.

[132] Borghi J., Cuevas S., Anton B., Iaia D., Gasparri G., Hanson M.A. (2024). Climate and health: a path to strategic co-financing?. Health Policy Plan.

[133] Weiler F. (2019). Adaptation and health: are countries with more climate-sensitive health sectors more likely to receive Adaptation aid?. Int J Env Res Public Health.

[134] Karva O., Jain R., Deo S. Mapping the literature on Development Assistance in Health: a Bibliometric analysis. New Delhi, India: observer Research Foundation; 2024. Accessed 1 May 2025. Available from: https://www.orfonline.org/research/mapping-the-literature-on-development-assistance-in-health-a-bibliometric-analysis.

[135] United Nations Environment Programme. Universal ownership – why environmental externalities matter to institutional investors, https://www.unepfi.org/industries/investment/universal-ownership-why-environmental-externalities-matter-to-institutional-investors-2/; 2011 [accessed 16 May 2024].

